# Politically Engaged Mindset of Everyday Coping in Relation to Nursing Values: A Phenomenological-Hermeneutic Study of District Nurses’ Experiences

**DOI:** 10.1177/23779608231157969

**Published:** 2023-02-19

**Authors:** Marianne Hauan, Kari Kvigne, Johanne Alteren

**Affiliations:** 1 Faculty of Nursing and Health Science, 1786 Nord University, Mo i Rana, Norway; 2 Faculty of Health and Social Science, 3207 Inland University of Applied Science, Elverum, Norway; 3 Faculty of Health Science and Social Care, 5562 Molde University College, Molde, Norway

**Keywords:** nursing values, everyday coping, political strategies, health promotion models

## Abstract

**Introduction:**

To accommodate challenges threatening the healthcare sector's sustainability, district nursing in Norway implemented the rehabilitative and health promoting mindset of everyday coping. When implementing new ideas and practices in nursing care, understanding the significance of this mindset on patient care and whether it corresponds to nursing values are important to ensure healthcare quality.

**Objective:**

This study aimed to understand how nurses practice care where everyday coping is implemented in district nursing and their experience of everyday coping as a mindset in relation to nursing values.

**Methods:**

A qualitative study was conducted including 19 observations and 19 narrative interviews with 10 district nurses, during two data collection periods. Data were analyzed using a phenomenological-hermeneutic method. The analysis process consisted of three steps: naïve reading, structural analysis, and comprehensive understanding.

**Results:**

The following two main themes and four sub-themes emerged from the data analyses: (i) Understanding individual patient situations; “Creating a nurse–patient relationship to understand the patient landscape” and “providing care based on individual patient needs,” (ii) knowing when and how to motivate or help patients; “distinction between motivating patients and causing stress” and “realistic and desirable demands to motivate patients to perform tasks.”

**Conclusion:**

Participants determined how to provide care to patients based on their values, professional knowledge, and individual patient situations. The patient landscape is diverse and everyday coping is unable to capture the diversity of patient groups. Thus, everyday coping is not expressed as an overall mindset in nursing practice.

## Introduction

Future challenges in the healthcare sector, such as the growing older population, the need for healthcare professionals, especially nurses, and significantly increasing expenses, threaten the sustainability of the healthcare sector (World Health Organization, 2022). To address these challenges, an increased focus on self-care strategies is being implemented into healthcare to keep the population as healthy as possible (World Health Organization, 2022) and in their own homes for as long as possible (Ministry of Health and Care Services, 2013, 2018). Norway implements self-care interventions in healthcare services through the Norwegian-developed mindset of everyday coping (Norwegian: hverdagsmestring) (Ministry of Health and Care Services, 2018). Everyday coping is defined as a mindset of rehabilitation and health promotion aimed at increasing patients’ self-care and geared toward achieving independence. For healthcare personnel and patients, this will mean increased focus on rehabilitation and health promotion in the form of guidance, education, and training patients in everyday tasks, such as guiding patients in performing personal hygiene or providing training and education to patients on blood close monitoring and performing injections themselves, such as insulin injections (Hartviksen & Sjølie, 2017; Ministry of Health and Care Services, 2018; Ness, 2016; Ness et al., 2012; Tuntland & Ness, 2014). In this paper, we illuminate nursing care in district nursing where everyday coping is implemented.

## Review of Literature

Even though everyday coping is implemented in several district nursing services in municipalities in Norway, there is limited research on everyday coping, with only one research report being found (Wittrock et al., 2020). The research report by Wittrock et al. (2020) conducted a survey on everyday coping in the home care service. The survey shows that 86% of health care personnel in home care (such as district nurses) report that they are working with everyday coping as a mindset in their daily work. The findings show that most health care personnel work with everyday coping in their daily work, yet there is limited research on how everyday coping affects the quality of care provided and experiences working according to everyday coping. This illustrates a significant gap in research regarding everyday coping as a mindset in health care services.

The theory of everyday coping states that activities should be based on patients’ own wishes and thoughts regarding meaningful activities (Tuntland & Ness, 2014; Wilde & Glendinning, 2012). Therefore, understanding what patients consider important is an essential part of everyday coping (Hartviksen & Sjølie, 2017; Ministry of Health and Care Services, 2018). However, everyday coping has been criticized for its excessive focus on the performance of physical tasks and its insufficient psychological understanding of important individual factors (Hauan et al., 2021). Professional, academic, and political discussions tend to focus on task-oriented everyday coping, such as nurses motivating patients to go out and get the mail or take out the rubbish (Hauan et al., 2021; Tuntland & Ness, 2014; Wittrock et al., 2020). However, this task-oriented approach in everyday coping is challenging from a nursing perspective (Vabø, 2018). Vabø (2008) accused the concept of everyday coping of being too narrow, as it focuses on the body and general management, falling short in consideration of the positives in everyday life. Furthermore, the focus on performing tasks may be improper for some patients, such as frail older adults with chronic disorders (Bakken, 2022). It is essential to remember that before they begin receiving district nursing assistance, older adults often undergo huge struggles to manage everyday activities (Bakken, 2022).

Coping has been defined as an experience of the strength to face challenges and feelings of having control over one's everyday life. Active and good coping helps you adapt to the new reality that enables you to see the differences between what you have to live with and what you yourself can change (Vifladt et al., 2004). This definition is concerned with patients’ subjective experiences of their situations, whereas everyday coping focuses more on the feasibility of performing tasks.

Research shows that patients receiving help from district nurses described meeting friendly nurses who took care of their integrity and showed respect (Moe et al., 2013). Furthermore, being seen as human beings in need of help was considered important for older adults and chronically ill patients to have the courage to meet challenges in daily life (Moe et al., 2013). In addition, patients reported that their relationship with nurses gave them the energy to continue and improve their physical and mental states (Strandås & Bondas, 2018). Integrity, respect toward people who require help, and building positive relationships are examples of nursing values that are challenged when new models or mindsets are implemented in nursing care, such as everyday coping.

Nursing values, such as nurse–patient relationships and helping patients cope with their illnesses, were established by the self-care deficit nursing theory (Orem et al., 2001). This theory defines self-care as activities that individuals initiate and perform on their own behalf to maintain life, health, and well-being, whereas nursing was defined as the act of assisting others in the provision and management of self-care to maintain or improve human functioning (Orem et al., 2001). This theory includes three systems of compensatory care in nursing: (1) wholly compensatory, (2) partly compensatory, and (3) educating and supporting. Further, care provision depends on individual patient situations (Orem et al., 2001).

New ideas and mindsets, such as everyday coping, in relation to nursing values must be discussed to develop nursing practice and theory (Tingen et al., 2009). Knowledge regarding the significance of newly implemented mindsets in patient care and whether they correspond to nursing values is important for providing quality healthcare. Furthermore, to our knowledge, there are limited research on how the mindset of everyday coping affect nurses’ daily work and their nursing values. Therefore, this study aimed to understand how nurses practice care where everyday coping is implemented in district nursing and their experience of everyday coping as a mindset in relation to nursing values.

## Methods

### Study Design

This study adopted an exploratory and descriptive design with a phenomenological-hermeneutical approach (Lindseth & Norberg, 2004; Ricoeur, 1976). The Consolidated Criteria for Reporting Qualitative Studies (COREQ) guided the study's reporting (Tong et al., 2007).

### District Nursing Context in the Study Area

District nursing in Norway provides comprehensive, coordinated, and flexible services that ensure continuity and allow patients to receive the services they need in their own homes at the appropriate time. The service has no age limit and patients receiving district nursing range from young children to older adults. Moreover, district nursing is not time-limited, and patients may receive healthcare from one visit to several years.

The municipality being studied was chosen as it has implemented everyday coping in district nursing. This municipality stretches over a geographical area of approximately 4,400 km and has about 26,000 inhabitants (Statistics Norway, 2020). Citizens reside both in the city center and in rural surroundings, including the coastline, valleys, and mountains. The municipality's strategy and direction document for 2014 stated that healthcare personnel, such as district nurses, should encourage patient self-care, specifically everyday coping.

When implementing everyday coping, the reablement service, which includes a physiotherapist, occupational therapist, and nurse, was responsible for the introduction of everyday coping to district nurses. The reablement service held lectures, created information brochures, and held meetings once every two weeks with district nurses. The lectures contained information on the benefits of working with everyday coping for patients and society, such as reduction in physical function impairment due to increased physical activity. Furthermore, the lectures included examples of everyday coping practice, which focused on practical examples, physical training, and everyday physical activities. Everyday coping was further introduced into district nursing through information brochures. The bi-weekly meetings focused on how to employ everyday coping as a mindset in individual patient situations.

District nurses in the studied municipality worked both day and evening shifts. At the start of every shift, they received a worklist prepared by the head nurse. The worklist was task-oriented and described patient assignments, including times and duration. The worklist assignments of district nurses included drug delivery, maintaining personal hygiene, wound care, blood sampling, catheterization, and blood glucose monitoring.

### Participants and Recruitment

District nurses were selected from a municipality in Norway where everyday coping was implemented. Initially, we approached the head nurse of a district nursing service verbally in person and in writing via email. The head nurse informed all of the 20 district nurses in the service about the study's purpose and procedure, distributed the written information regarding the study, discussed relevant ethical issues, and asked for volunteers. Furthermore, participants were informed regarding the opportunity to withdraw their participation at any time without any explanation. A total of 14 district nurses agreed to participate; however, four were excluded because they did not have a permanent position in the district nursing service. Therefore, ten district nurses (8 women and 2 men) with 10–18 years of experience were included in the study.

### Data Collection

Observations and interviews were collected by the first author during two periods: September–October 2016 and February–March 2017. Of the ten district nurses included in the study, nine participated in the first period and ten in the second period. Thus, the data consisted of 19 observations and 19 interviews.

Observations were conducted before interviews and took place during the first half of the nurses’ workday, from 8 am to 11.30 am. The observations aimed to gain a better understanding of district nursing practice with everyday coping and nurses’ experiences with everyday coping as a mindset in relation to nursing values.

Observation notes were compiled following the observations and comprised descriptions of nursing practice care in patient situations. These notes were used during analysis. These observations created shared experiences, providing an in-depth understanding of district nursing practice. Interviews were conducted after observations and lasted 18–65 min (mean 36.2 min), as response length varied. To obtain a comprehensive description, the interviews were unstructured and conducted as a dialogue (Brinkmann & Kvale, 2015) designed to capture the nursing practice in district nursing, where everyday coping is implemented. Interviews were based on observations and reflected on nurses’ experiences in district nursing practice and their values as professional nurses. The interviews were digitally recorded, transcribed verbatim, and anonymized.

### Ethical Considerations

The study was approved by the Regional Committee for Medical and Health Research Ethics and the Norwegian Center for Research Data. Nurses and patients involved in the study provided written informed consent to participate. Patients were included in the observations; however, no personal information was collected. Nurses and patients were informed that they could withdraw from the study at any time without any consequences. The anonymity of the participants was ensured.

### Data Analysis

Narrative interviews were analyzed using the phenomenological-hermeneutical method (Lindseth & Norberg, 2004), which was inspired by a combination of hermeneutics and phenomenology (Ricoeur, 1976). This approach was used to provide insight into district nursing practice with everyday coping and nurses’ experiences with everyday coping as a mindset in relation to nursing values.

Interviews were analyzed using three steps: naïve reading, structural analyses, and comprehensive understanding (interpreted whole). This alternated the focus between the whole and parts, referred to as the hermeneutical circle (Lindseth & Norberg, 2004).

Naïve reading involves reading the interviews and observation notes several times to grasp their meaning as a whole. Naïve reading revealed significant diversity in patient groups and situations (i.e., the patient landscape) in district nursing. Furthermore, nurses highlighted the importance of understanding the patient landscape in deciding nursing procedures in individual situations. Moreover, participants reported the importance of finding a balance between encouraging patients to engage in activity and performing some level of compensatory care (helping them).

Thematic structural analysis involves methodological interpretation of the text to identify and formulate themes (Table 1). The first part of the structural analysis process is to read and divide the whole interview text into meaning units. A meaning unit can be a part of a sentence, a sentence, or several sentences that convey a meaning (Lindseth & Norberg, 2004). Then the meaning unit is reflected in relation to the naïve reading, and the meaning unit is condensed to capture its essential meaning as concisely as possible. Based on the condensed meaning units, sub-themes and themes are formed. The themes are then reflected on in relation to the naïve reading (Lindseth & Norberg, 2004).

**Table 1.** Examples of Structural Analysis, Sub-Themes, and Main Themes.

Meaning unit	Condensed form of meaning unit	Sub-theme	Main theme
In my opinion, nurses always consider their actions and the best interests of patients.	Nurses always consider their actions and patients’ best interests.	Providing care based on individual patient needs.	Understanding individual patient situations.
Occasionally, I can see when a patient needs more time to perform tasks one day, I can see that because I have worked here so long that I know most patients very well.	Occasionally, some patients need more time to perform tasks. I can see that because I know most patients well.	Creating a nurse–patient relationship to understand the patient landscape.	Understanding individual patient situations.
If we (the nurses) are very strict, the quality of care can be poor, such as if we do not see or understand the whole patient situation and demand that patients do things themselves that they cannot manage. Sometimes we have to help the patients.	The quality of care can be poor if we do not understand the whole patient situation, and demand patients to do things they don’t manage. Sometimes we need to help.	Distinction between motivating patients and causing stress.	Importance of knowing when and how to motivate or help patients.
I think it is important that nurses have realistic demands and expectations of the patients that match their abilities.	Realistic demands and expectations that match patients’ abilities are important.	Realistic and desirable demands to motivate patients to perform tasks.	Importance of knowing when and how to motivate or help patients.

Comprehensive understanding involves a summary of the main themes and sub-themes in relation to the research question and context of the study (Lindseth & Norberg, 2004). After reading the analysis, all authors discussed the results until they reached an agreement.

## Results

The results revealed two themes: (i) understanding individual patient situations and (ii) the importance of knowing when and how to motivate or help patients (Table 2).

**Table 2.** Overview of the Results.

Sub-theme	Main theme
Providing care based on individual patient needs.	Understanding individual patient situations.
Creating a nurse–patient relationship to understand the patient landscape.	
Distinction between motivating patients and causing stress.	Importance of knowing when and how to motivate or help patients.
Realistic and desirable demands to motivate patients to perform tasks.	

### Understanding Individual Patient Situations

Observation notes and nurses’ narratives revealed diversity among patient groups and the need for healthcare in district nursing. Healthcare included help with compression stockings, help with personal hygiene, supervision to update medication lists from the patient's physician, intravenous infusion, and parenteral nutrition. Nurses handled these situations differently depending on the patient and procedure. Several nurses said that they always considered the patient's situation before determining how to provide care. Furthermore, nurses’ actions were shaped by the patient's situation rather than the everyday coping mindset.

*I do not want to work on a matter of principle. Even if a person is overweight and benefits from being active and involved in performing tasks, I will not say that they have to walk to the kitchen so that we can cook together. Rather, we must consider the psychological situation: why does the patient not walk to the kitchen, what makes the patient not want to walk?*

Through the interviews, several nurses highlighted the importance of understanding the patient landscape to provide good care. To fully understand a patient's landscape, it is essential to establish a good nurse–patient relationship, which enables nurses to build an emotional connection with their patients and make thorough observations. One participant reported an incident in which they did a patient check-up regarding recurrent depressive periods. When the nurse came to the patient's home, they observed that the kitchen was dirty and had not been cleaned for a while. The patient was sitting on the couch staring out into the garden at a bird feeder where birds were eating. The nurse explained that, based on the patient's depressed posture, the way he talked, and the mess in the apartment, it was apparent that the patient was in a severe depressive period. Rather than encouraging the patient to wash the kitchen, which is a classic everyday coping technique, the nurse sat down and talked to the patient about the birds in the bird feeder and birds arriving with the coming of spring. The conversation lasted approximately 5 min. The next day, the same nurse returned to the patient with depression for his daily check-up. The patient was still scarcely talking but had a more uplifted posture, wore clean clothes, and cleaned the kitchen. The nurse considered this a sign that the patient's depressive state improved. Moreover, the nurse emphasized that this was difficult to detect without good knowledge of the patient's situation and behavior.

### Importance of Knowing When and How to Motivate or Help Patients

One of the nurses reported that it is important for someone to make demands and set expectations of patients, as it is difficult for them to motivate themselves. Therefore, nurses’ expectations can drive patients to be more involved and active in performing daily tasks. However, expectations must be realistic and match the patient's desires. If the patient does not see the importance of the activity or expectations are unachievable, this may have the opposite effect and weaken the patient's sense of coping.

However, the nurses reported that in some situations, the patients became annoyed and were dissatisfied with the nurses when they encouraged the patient to be more involved and active. In these situations, nurses stated that it was hard to comprehend when to continue motivating patients to take part in an activity and when to not coax the patient, particularly when the patient was disinterested or was not capable of more. Situations where the nurse tried to motivate patients to be active, but the patient did not show any enthusiasm, were challenging and frustrating for the nurses.

*It is hard to try to motivate day after day, knowing that the patient's life would be easier if the patient just wanted to do something about it* *…* *You may see that the patient is getting it for a while, but then they go back to the old pattern, and you are thinking: how long should I keep doing this?*

Moreover, nurses stated that it is important to be aware of the patient's whole situation. Patients may have various reasons to not perform tasks, and their conditions may differ daily. Patients may be active for several days and do little on other days. This may be due to physical or psychological reasons. In addition, the nurses reported that as they knew their patients well, they could easily observe when their patients have bad days and accordingly adapt their care to meet patient needs. Nurses highlighted the importance of remembering that patients with help from district nurses need compensatory care and to not lose focus on what they need help with. Furthermore, the nurses stressed that it is important to find a good balance between motivating patients to be active and compensating where needed. Even if the patient is doing something today, they may not be able to do it tomorrow. Some nurses said that if a patient had a bad day, they spent more time with that patient and tended to focus more on helping them rather than motivating them to be more active in performing tasks.

## Discussion

This study indicated that nurses provide care based on patients’ situations and nursing values rather than the principle of everyday coping. This suggests that everyday coping does not function as an overall mindset among nurses but rather as a consideration if useful in the individual patient situation. In particular, when working with older adults and frail people, being too harsh on them and forcing them to independently do things that they do not want to or are not capable to do can be perceived as offensive or insulting (Bakken, 2022). This may challenge professional nurses’ values, such as patients’ well-being. However, working according to the patient landscape and not strictly following the everyday coping mindset may conflict the political goal, set by the government and implemented by the municipality, of everyday coping to create more self-reliant patients and reduce resource pressure on the healthcare sector (Ministry of Health and Care Services, 2018).

Although health-promoting, preventive, and rehabilitative work is an important part of nursing practice, nursing values include more than merely working toward patients’ abilities to recover or be self-reliant. In addition, nursing values involve compensatory care and taking care of sick people who do not recover (Orem et al., 2001). Nurse–patient relationships are an essential nursing value; high quality and trust are required to provide professional nursing care (Wiechula et al., 2016). Furthermore, nurses who follow up with and spend time with their patients, including helping patients deal with existential pain and loneliness, are essential for high-quality care (Strandås & Bondas, 2018). Patients’ dedication to nurses helped nurses detect needs or problems of patients, which helped support patients’ efforts to cope independently (Strandås & Bondas, 2018). Moreover, a good nurse–patient relationship may facilitate patients’ adaptation to their disease, alleviate their suffering, and enable them to find meaning in their suffering (Mok & Chiu, 2004). Using their unique competence and healthy relationships with patients, nurses seek in-depth knowledge and understanding of patient's physical, cognitive, and psychosocial conditions as aids in choosing the right level of assistance when tailoring care, which is an essential part of nursing values (Orem et al., 2001; Strandås & Bondas, 2018). This is where everyday coping falls short, and the lack of nuances in performing everyday coping becomes visible in complex nursing practice. Although everyday coping is universally designed and should fit all healthcare services, the mindset does not consider the patient's landscape as a whole, particularly for those with chronic progressive diseases. With such patients, nurses cannot focus only on patients’ abilities to perform everyday tasks, and rehabilitative goals are often not realistic. Furthermore, finding a balance between motivating patients to be active and providing compensatory care is challenging with everyday coping. This may suggest that the mindset of everyday coping needs to be more nuanced in its practical use, such as the three-level nursing system that describes the amount of help necessary based on patient needs (Orem et al., 2001). This nuanced model considers that patients are different and are in different situations and conditions (Orem et al., 2001). Nurses work based on their values by evaluating the patient situation and performing tailor-made compensatory care. They may motivate and guide the patient on a supportive-educative level one day and provide partially compensatory care to the same patient when they need more help. Therefore, the lack of a nuanced theory regarding everyday coping is challenging for nursing values. If nurses fail to follow their values to provide good patient care, their position as professional nurses may be threatened (Angel & Vatne, 2017).

In addition, the present findings revealed that the concept of “coping” in everyday coping is too narrow and lacks the nuance required to correspond with nurses’ values. Coping in everyday life is more than merely being able to perform tasks; therefore, individually important factors are not only task-oriented duties (Hauan et al., 2021). In district nursing, important factors for patients may change daily and may not be related to everyday coping, as exemplified by the story of the patient observing a birdfeeder. A more nuanced definition of coping (Vifladt et al., 2004) is more in line with nursing values, as it considers patients’ current experiences and not only their ability to perform everyday tasks.

Frail older adult patients have a long life behind them and a short life in front of them, and living well may not necessarily involve self-managing and goal-setting (Bakken, 2022). Rather, it may include getting help and support in everyday activities, allowing them to focus their energy on what is important to them. Nursing values seek to provide patients with security and, as much as possible, a life free from ailments and discomfort (Normann et al., 2006). Nursing values involve assisting patients in establishing and managing self-care to maintain or improve human functioning (Orem et al., 2001). Therefore, although the politically engaged mindset of everyday coping is implemented in district nursing, nurses did not utilize it in scenarios where it conflicted with nursing values. Nursing values took precedence over the political guidelines of everyday coping.

### Strengths and Limitations

This qualitative study strived to ensure trustworthiness by providing a sufficient description of nurses’ experiences with everyday coping as a mindset in patient care.

During the interviews, the researcher summarized the main objectives of the dialogue to ensure that the content was correctly understood (Sandelowski & Barroso, 2002).

However, this study has some limitations. Only one geographical area in the studied municipality (i.e., two district nurse services) was included. However, this led to rich, in-depth, and detailed interviews and observations on district nurses’ experiences with everyday coping in patient situations.

All nurses who participated in the study attended lectures of the reablement service on working with everyday coping as a mindset. Thus, district nurses had knowledge of the mindset before they were observed and interviewed.

All researchers participated in the analysis process, which strengthened the validity of the analysis process. The data collection method included stories that were in line with the study's purpose and goals. The analysis was in line with the scientific theory of this method.

## Implications for Practice

This study provided insight into nurses’ experiences related to working in district nursing, where the mindset of everyday coping is implemented. Thus, this study will contribute to developing and strengthening professional nursing in community services. Knowledge regarding nursing practices where everyday coping is implemented is important to healthcare management, politicians, and the nursing profession when planning future healthcare. A politically desired mindset to make patients more self-reliant (e.g., everyday coping) in nursing care may lead to some challenges for nursing practice and may not have the politically desired effect on patients’ ability to self-care.

## Conclusions

This study aimed to gain knowledge of how nurses practice when everyday coping is implemented in district nursing and their experience of everyday coping as a mindset in relation to nursing values. Our results highlighted that nurses decided how to provide care for their patients based on their knowledge and nursing values in the individual patient situation. In addition, the patient landscape was diverse and everyday coping was too narrow to comprehend the diversity of patient groups. Thus, everyday coping is not expressed as an overall mindset in nursing performance.

Furthermore, working from a task-oriented and patient-involvement perspective is a political strategy and is more common in community services. Therefore, further studies are required to expand knowledge regarding the consequences of these perspectives on patients and for the future development of healthcare and nursing practice.
